# Synthesis of Oligonucleotides Containing 2′-*N*-alkylaminocarbonyl-2′-amino-LNA (2′-urea-LNA) Moieties Using Post-Synthetic Modification Strategy

**DOI:** 10.3390/molecules25020346

**Published:** 2020-01-15

**Authors:** Shoko Yamashita, Kodai Nishida, Takashi Osawa, Ayumi Nakanishi, Yuta Ito, Yoshiyuki Hari

**Affiliations:** 1 Faculty of Pharmaceutical Sciences, Tokushima Bunri University, Nishihama, Yamashiro-cho, Tokushima 770-8514, Japan; 2 Graduate School of Pharmaceutical Sciences, Osaka University, 1-6 Yamadaoka, Suita 565-0871, Japan

**Keywords:** bridged nucleic acid, post-synthetic modification, modified oligonucleotides, 2′-urea-LNA, UV-melting experiment

## Abstract

The post-synthetic modification of an oligonucleotide is a powerful strategy for the synthesis of various analogs of the oligonucleotide, aiming to achieve the desired functions. In this study, we synthesized the thymidine phosphoramidite of 2′-*N*-pentafluorophenoxycarbonyl-2′-amino-LNA, which was introduced into oligonucleotides. Oligonucleotides containing a 2′-*N*-pentafluorophenoxycarbonyl-2′-amino-LNA unit could be isolated under ultra-mild deprotection conditions (50 mM K_2_CO_3_ in MeOH at room temperature for 4 h). Moreover, by treatment with various amines as a post-synthetic modification, the oligonucleotides were successfully converted into the corresponding 2′-*N*-alkylaminocarbonyl-2′-amino-LNA (2′-urea-LNA) derivatives. The duplex- and triplex-forming abilities of the synthesized oligonucleotides were evaluated by UV-melting experiments, which showed that 2′-urea-LNAs could stabilize the nucleic acid complexes, similar to the proto-type, 2′-amino-LNA. Thus, 2′-urea-LNAs could be promising units for the modification of oligonucleotides; the design of a substituent on urea may aid the formation of useful oligonucleotides. In addition, pentafluorophenoxycarbonyl, an amino moiety, acted as a precursor of the substituted urea, which may be applicable to the synthesis of oligonucleotide conjugates.

## 1. Introduction

Chemically modified oligonucleotides have been widely used in areas such as nanotechnology and drug development. The purpose of such chemical modification is to realize the desired functions depending on the specific applications. The functions of small molecules can be explored using a large number of derivatives; however, this is not easy for many modified oligonucleotides because of their synthetic difficulty. The preparation of a modified oligonucleotide is time-consuming as it involves these processes—(i) synthesis of the modified building block, and (ii) synthesis of the oligonucleotide including the building block employing an oligonucleotide synthesizer. Under these circumstances, chemical modification following the synthesis of the oligonucleotide—called “post-synthetic modification”—is a powerful strategy enabling us to prepare various derivatives from a single oligonucleotide encompassing a reactive site [1,2,3,4,5,6,7,8].

**Figure 1:**
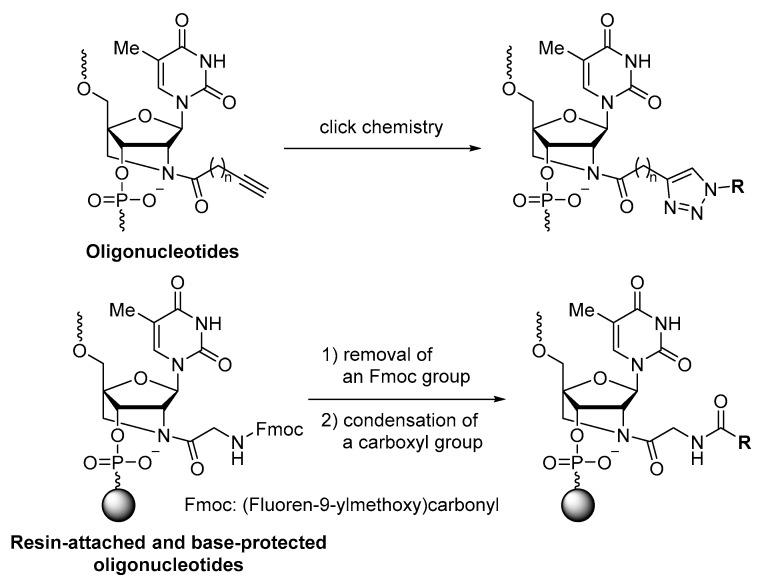
Synthesis of 2′-*N*-substituted 2′-amino-LNA in oligonucleotides by post-synthetic modification using click chemistry and amidation.

Moreover, bridging between the 2′- and 4′-positions of the furanose ring has been actively studied as a sugar modification technique for oligonucleotides. The conformational restriction of the sugar and the bulkiness of the bridge moiety are expected to improve the hybridizing ability of the oligonucleotides to target nucleic acids and reduce nuclease degradation [9,10,11,12,13]. In particular, 2′-amino-LNA, a 2′,4′-bridged nucleic acid, can have various substituents of the 2′-amino group [14]; therefore, 2′-amino-LNA would be a useful scaffold to explore oligonucleotides possessing the desired properties. Previous studies have reported oligonucleotides containing 2′-*N*-substituted 2′-amino-LNA derivatives, such as 2′-*N*-alkyl, 2′-*N*-acyl, and 2′-*N*-alkoxycarbonyl derivatives [15,16,17,18,19,20,21,22]. In general, the synthesis was based on a common method using each modified phosphoramidite; however, post-synthetic approaches using click chemistry [20,21,23] and amidation [24] were also applied to the synthesis of the 2′-*N*-substituted 2′-amino-LNA derivatives in the oligonucleotides (Figure 1). The substrates containing the reactive sites are somewhat specific, and the 1,2,3-triazole and glycyl units remain after post-synthetic modification. Thus, the development of a new post-synthetic modification method for the 2′-*N*-substituted 2′-amino-LNA is essential.

We considered that 2′-amino-LNA bearing an active carbamate, like a pentafluorophenyl carbamate, could be converted into 2′-*N*-alkylaminocarbonyl-2′-amino-LNA (2′-urea-LNA) via the post-synthetic treatment with amines. With this method, various amines that are commercially available or easily synthesized can be used and the procedure is simple to perform (amine treatment). Moreover, urea is the only unit that remains on the oligonucleotide. We synthesized oligonucleotides containing various 2′-urea-LNA derivatives using post-synthetic modification and evaluated their duplex- and triplex-forming ability. The details are described herein.

## 2. Results and Discussion

### 2.1. Synthesis

**Scheme 1:**
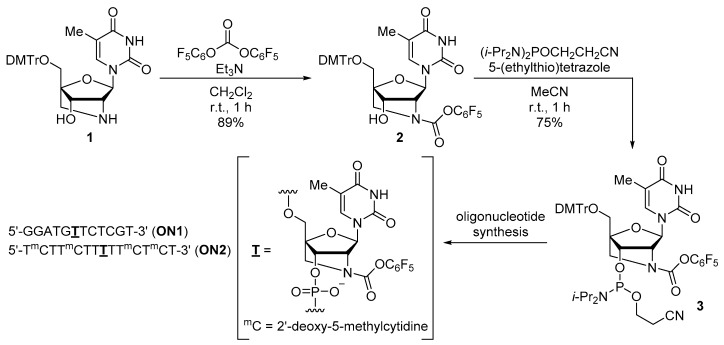
Synthesis of thymidine phosphoramidite **3** and modified oligonucleotides.

The synthesis of thymidine phosphoramidites with various 2′-*N*-alkoxycarbonyl-2′-amino-LNA modifications was previously reported by us [22]. Thus, according to the procedure, a thymidine phosphoramidite with 2′-*N*-pentafluorophenoxycarbonyl-2′-amino-LNA modification was synthesized as shown in Scheme 1. Compound **1** was treated with bis(pentafluorophenyl) carbonate in the presence of Et_3_N to produce the 2′-*N*-pentafluorophenoxycarbonyl derivative (**2**) in 89% yield. In this reaction, no 3′-*O*-pentafluorophenoxycarbonyl derivative was obtained, unlike the case of other alkoxycarbonyl derivatives [22]. This was probably because of the poor stability of the 3′-*O*-pentafluorophenoxycarbonyl derivative, resulting from the good leaving ability of the pentafluorophenoxy group. Phosphitylation of **2** using (*i*-Pr_2_N)_2_POCH_2_CH_2_CN and 5-(ethylthio)tetrazole afforded the desired phosphoramidite (**3**), which is a suitable building block for the synthesis of oligonucleotides, in 75% yield.

Next, we synthesized the oligonucleotides using common phosphoramidite chemistry on an oligonucleotide synthesizer; the sequences of the oligonucleotides are shown in Scheme 1. Phenoxyacetyl (Pac) and isopropylphenoxyacetyl (*i*-PrPac) protections were used for the dA and dG phosphoramidites, respectively. Furthermore, the nucleobases in dC and 2′-deoxy-5-methylcytidine (d^m^C) phosphoramidites were acetyl-protected. The coupling time was increased from 25 s to 10 min when phosphoramidite (**3**) was introduced into the oligonucleotides, and the coupling efficiency was estimated to be over 95%, based on the trityl monitoring observed in the removal of the 5′-DMTr group. After the synthesis of the oligonucleotides on a DNA synthesizer, the fully protected oligonucleotides attached to control pore glass (CPG) resin were subjected to ultra-mild conditions (50 mM K_2_CO_3_ in MeOH at room temperature for 4 h) to produce the corresponding 5′-*O*-DMTr-oligonucleotides via the removal of the cyanoethyl groups in the phosphotriester moieties and the protecting groups in nucleobases, followed by cleavage from the resin. The 2′-*N*-pentafluorophenoxycarbonyl unit was tolerant of the base treatment. The DMTr-removal and purification step yielded the desired oligonucleotides modified with 2′-*N*-pentafluorophenoxycarbonyl-2′-amino-LNA.

**Scheme 2:**
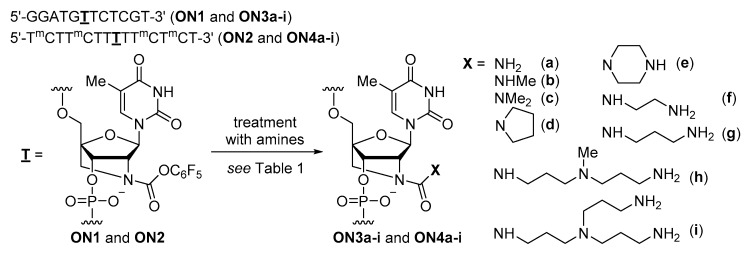
Synthesis of modified oligonucleotides **ON3a**–**i** and **ON4a**–**i** by post-synthetic modification.

**Figure 2:**
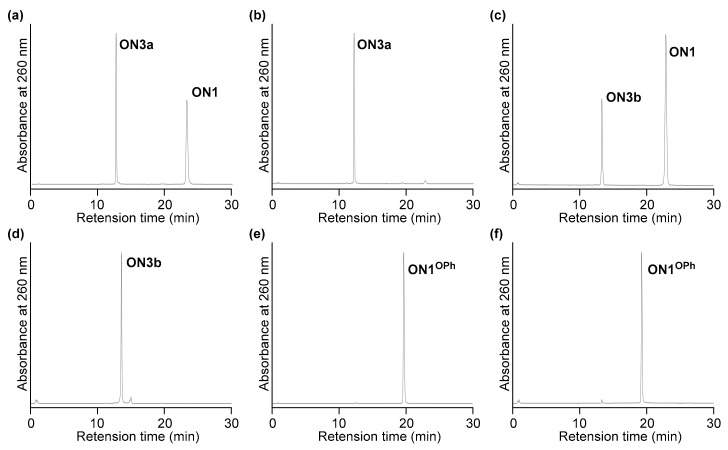
HPLC profiles of the crude products after post-synthetic modification: (**a**) Treatment of **ON1** with 10 M NH_3_ aq. at 30 °C for 4 h; (**b**) Treatment of **ON1** with 10 M NH_3_ aq. at 30 °C for 24 h; (**c**) Treatment of **ON1** with 0.1 M MeNH_2_ aq. at 30 °C for 2 h; (**d**) Treatment of **ON1** with 0.5 M MeNH_2_ aq. at 30 °C for 4 h; (**e**) Treatment of **ON1^OPh^** with 10 M NH_3_ aq. at 30 °C for 24 h; (**f**) Treatment of **ON1^OPh^** with 0.5 M MeNH_2_ aq. at 30 °C for 24 h.

The 2′-*N*-Pentafluorophenoxycarbonyl-2′-amino-LNA was converted within an oligonucleotide (**ON1**) by the treatment with various amines (Scheme 2 and Table 1). The treatment with 10 M NH_3_ aq. at 30 °C for 4 h left the unreacted oligonucleotide (**ON1**), although the production of the corresponding unsubstituted 2′-urea-LNA was also observed (**ON3a**) (Figure 2a). The prolonged reaction time to 24 h yielded the desired **ON3a** with high efficiency, and it was isolated in 76% yield (Figure 2b). More than half of the **ON1** remained in the treatment with 0.1 M MeNH_2_ aq. at 30 °C for 2 h (Figure 2c). **ON1** almost disappeared at an increased concentration of MeNH_2_ aq. to 0.5 M. Finally, **ON1** was treated with 0.5 M MeNH_2_ aq. at 30 °C for 4 h to produce the desired methylurea **ON3b** (Figure 2d) in 86% yield, without any unreacted **ON1** left. The use of 0.5 M Me_2_NH aq. as a secondary amine successfully gave the dimethylurea (**ON3c**) in 68% yield. When pyrrolidine, piperazine, ethylenediamine, 1,3-propanediamine, 3,3′-diamino-*N*-methyldipropylamine, and tris(3-aminopropyl)amine were used, oligonucleotides containing the corresponding 2′-urea-LNA with the respective substituents on the urea moieties were obtained. These results suggested that 2′-*N*-pentafluorophenoxycarbonyl-2′-amino-LNA was a good precursor for the construction of 2′-amino-LNA analogs with a substituted urea unit by post-synthetic modification. In contrast, when an oligonucleotide containing 2′-*N*-phenoxycarbonyl-2′-amino-LNA (**ON1^OPh^**) was treated with 10 M NH_3_ aq. at 30 °C for 24 h and 0.5 M MeNH_2_ aq. at 30 °C for 24 h, almost no reaction was observed in all cases (Figure 2e,f); this could be due to the low reactivity of the phenyl carbamate.

A 14-mer homopyrimidine oligonucleotide (**ON2**) was converted into oligonucleotides (**ON4a–i**) containing the substituted analogs of 2′-urea-LNA under the same conditions (Scheme 2 and Figure S1 (Supplementary Materials)). The isolated yields are shown in Table 1.

### 2.2. Evaluation

**Figure 3:**
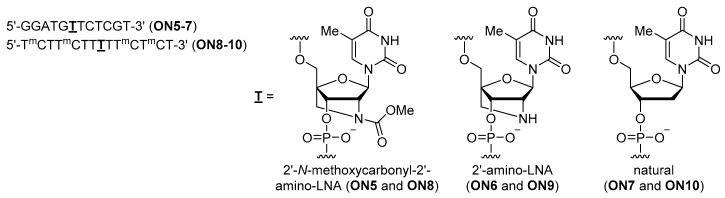
Oligonucleotides containing 2′-(methoxycarbonyl)amino-LNA and 2′-amino-LNA, and natural oligonucleotides used in this study.

**Figure 4:**
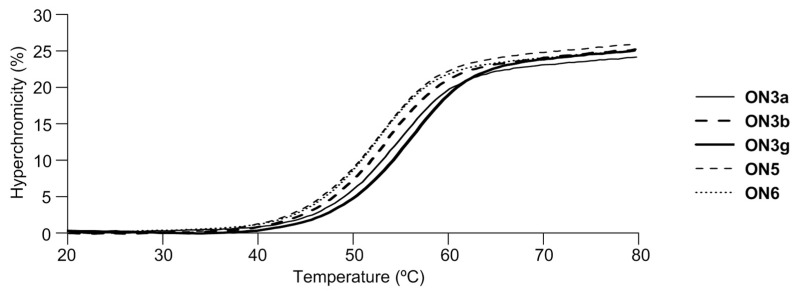
Representative UV-melting profiles of the duplexes formed with ssDNA and modified oligonucleotides.

UV-melting experiments of duplexes between 12-mer oligonucleotides (**ON3a**–**i**) containing 2′-urea-LNA analogs and single-stranded DNA (ssDNA) or ssRNA was performed; the obtained melting temperatures (*T*_m_) were compared to those of 2′-(methoxycarbonyl)amino-LNA (**ON5**) (as a reference of 2′-*N*-substituted 2′-amino-LNA), unsubstituted 2′-amino-LNA (**ON6**), and natural (**ON7**) (Table 2 and Figure 3 and Figure 4). Increasing the number of substituents on the urea moiety of 2′-urea-LNA tended to decrease the stability of duplexes with ssDNA; the *T*_m_ values of unsubstituted (**ON3a**), methylurea (**ON3b**), dimethylurea (**ON3c**), and pyrrolidinocarbonylamine (**ON3d**) were 54 °C, 53 °C, 52 °C and 52 °C, respectively. Moreover, the disubstituted ureas **ON3c** and **ON3d** had the same hybridizing ability to ssDNA as 2′-(methoxycarbonyl)amino-LNA (**ON5**) and the parent 2′-amino-LNA (**ON6)**, which suggested that the 2′-urea unit favored the formation of the duplex with ssDNA. The introduction of an amino group into the *N*-substituents of urea stabilized the duplexes. For example, the *T*_m_ of aminoethyl urea (**ON3f**) and aminopropyl urea (**ON3g**) (55 °C) was slightly higher than that of the same monosubstituted methylurea (**ON3b**) (53 °C). No further stabilization occurred in **ON3h** and **ON3i** which contained more amino groups.

In the case of duplex formation with ssRNA, although **ON3i** (bearing a branched bis(aminopropyl)amino group) showed a decreased *T*_m_ (55 °C), the stabilization abilities by other 2′-urea-LNA derivatives were comparable to that by carbamate (**ON5**) or unsubstituted 2′-amino-LNA (**ON6**). For all 2′-urea-LNA derivatives, the duplexes were significantly stabilized when compared with the natural duplex by **ON7**. The results implied that a linear long chain on the urea unit might not influence the stability of the duplex formed with ssRNA.

**Figure 5:**
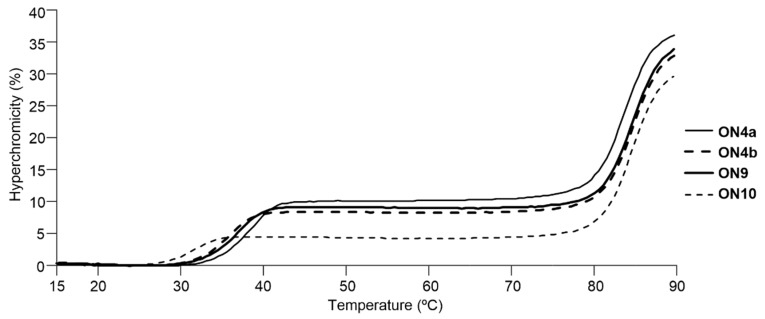
Representative UV-melting profiles of the triplexes formed with dsDNA and modified oligonucleotides.

UV-melting experiments of triplexes between 14-mer oligonucleotides (**ON4a**–**i**) containing 2′-urea-LNA analogs and hairpin dsDNA were also performed (Figure 5 and Table 3). Triplexes formed by oligonucleotides containing 2′-urea-LNA derivatives were stable analogous to those by methoxycarbonyl (**ON8**) and 2′-amino-LNA (**ON9**), though 2′-urea-LNA with a pyrrolidine unit (**ON4d**) decreased the stability of the triplex significantly.

## 3. Materials and Methods

### 3.1. General

All moisture-sensitive reactions were conducted in well-dried glassware under an Ar atmosphere. Anhydrous CH_2_Cl_2_ and MeCN were used as purchased. ^1^H-NMR, ^13^C-NMR and ^31^P-NMR spectra were recorded on a Bruker AVANCE III HD 500 MHz spectrometer equipped with a BBO cryoprobe, and an Agilent/Varian 400 MHz spectrometer. The chemical shift values were reported in ppm, relative to the internal tetramethylsilane (δ = 0.00 ppm) or solvent residual signals (δ = 3.31 ppm for CD_3_OD) for ^1^H-NMR, solvent residual signals (δ = 77.0 ppm for CDCl_3_ and δ = 49.0 ppm for CD_3_OD) for ^13^C-NMR, and external 5% H_3_PO_4_ (δ = 0.00 ppm) for ^31^P-NMR. High-resolution mass spectrometry was performed on a Waters SYNAPT G2-Si (Quadrupole/TOF). For column chromatography, silica gel PSQ-60B (Fuji Silysia) was used. The progress of the reaction was monitored by analytical thin-layer chromatography (TLC) on pre-coated aluminum sheets (Silica gel 60 F254 by Merck). For HPLC, a JASCO EXTREMA (PU-4180, CO-4060 or CO-4061, UV-4075, and AS-4050) instrument with a CHF122SC (ADVANTEC) fraction collector was used. UV-melting experiments were carried out using a JASCO V-730 UV/VIS spectrophotometer equipped with a *T*_m_ analysis accessory. The synthesis of oligonucleotides was performed on an automated DNA synthesizer (Gene Design nS-8II).

### 3.2. Synthesis

Compound **2**: Bis(pentafluorophenyl) carbonate (152 μL, 1.1 eq.) was added to a solution of compound **1** [22] (200 mg, 0.35 mmol) and Et_3_N (73 μL, 1.5 eq.) in anhydrous CH_2_Cl_2_ (5 mL) at 0 °C, under an argon atmosphere. After stirring at room temperature for 1 h, sat. NaHCO_3_ aq. was added to the reaction mixture. After dilution with AcOEt, the organic layer was washed with sat. NaHCO_3_ aq., H_2_O, and sat. NaCl aq., and dried over Na_2_SO_4_. The solvent was evaporated, and the residue was purified by silica gel column chromatography (hexane/AcOEt 1:1) to afford compound **2** (244 mg, 89%) as a pale yellow powder. Compound **2** was shown to exist as a mixture of carbamate rotamers by NMR spectroscopy (Figure S2).

^1^H-NMR (CDCl_3_): δ 1.60 (1.5H, s), 1.62 (1.5H, s), 2.43 (0.5H, brs), 3.11 (0.5H, brs), 3.49–3.77 (4H, m), 3.80 (3H, s), 3.80 (3H, s), 4.36 (0.5H, s), 4.38 (0.5H, s), 4.81 (0.5H, s), 4.83 (0.5H, s), 5.65 (0.5H, s), 5.70 (0.5H, s), 6.85–6.87 (4H, m), 7.28–7.47 (9H, m), 7.53 (0.5H, s), 7.66 (0.5H, s), 8.45 (0.5H, brs), 8.58 (0.5H, brs). ^13^C-NMR (CDCl_3_): δ 12.40, 12.54, 52.44, 52.71, 55.17, 55.21, 58.94, 59.35, 63.76, 64.13, 69.26, 69.81, 86.80, 86.86, 86.93, 88.09, 88.71, 110.18, 110.46, 113.29, 113.30, 113.33, 113.36, 123.92, 125.18–125.52 (m), 127.70, 127.18, 127.98, 128.00, 128.06, 129.99, 130.01, 130.04, 130.05, 134.29, 134.57, 135.14, 135.19, 136.40, 136.61–136.82 (m), 138.33–138.84 (m), 140.23–140.26 (m), 142.56–142.69 (m), 144.22, 144.40, 149.32, 149.81, 150.35, 150.84, 150.88, 158.69, 158.75, 163.73, 164.02. HRMS (ESI-TOF): Calcd. for C_39_H_32_F_5_N_3_NaO_9_ [MNa]^+^ 804.1956, found 804.1957.

Compound **3**: 5-ethylthiotetrazole (25 mg, 1.0 eq.) was added to a solution of compound **2** (150 mg, 0.19 mmol) and (*i*-Pr_2_N)_2_POCH_2_CH_2_CN (122 μL, 2.0 eq.) in anhydrous MeCN (3 mL) at 0 °C, under an argon atmosphere. After stirring at room temperature for 1 h, sat. NaHCO_3_ aq. was added to the reaction mixture. After dilution with AcOEt, the organic layer was washed with H_2_O and sat. NaCl aq., and dried over Na_2_SO_4_. The solvent was evaporated, and the residue was purified by silica gel column chromatography (hexane/AcOEt 1:1) to afford compound **3** (140 mg, 75%) as a white powder. Compound **3** was shown to exist as a mixture of carbamate rotamers by NMR spectroscopy (Figure S3).

^1^H NMR (CDCl_3_): δ 0.99–1.17 (12H, m), 1.52–1.56 (3H, m), 2.38–2.42 (1H, m), 2.53–2.63 (1H, m), 3.44–3.76 (8H, m), 3.79–3.81 (6H, m), 4.49–4.54 (1H, m), 4.89–5.04 (1H, m), 5.73–5.74 (1H, m), 6.83–6.88 (4H, m), 7.25–7.46 (9H, m), 7.65–7.69 (1H, m), 8.56 (1H, brs). ^31^P-NMR (CDCl_3_): δ 149.60, 149.73, 149.84. HRMS (ESI-TOF): Calcd. for C_48_H_49_F_5_N_5_NaO_10_P [MNa]^+^ 1004.3035, found 1004.3035.

Oligonucleotides **ON1** and **ON2**: 2′-*N*-Pentafluorophenoxycarbonyl-2′-amino-LNA-T phosphoramidite **3**, dA(Pac)-phosphoramidite, dG(*i*Pr-Pac)-phosphoramidite, dC(Ac)-phosphoramidite, dT-phosphoramidite, and d^m^C(Ac)-phosphoramidite were used in this process. The syntheses of these oligonucleotides were performed on a 0.2 μmol scale using a standard phosphoramidite protocol (DMTr-ON mode), except for the phosphoramidite **3**, which had a prolonged coupling time of 10 min. Cleavage from the CPG support and removal of the protecting groups were accomplished using 50 mM K_2_CO_3_ in MeOH at room temperature for 4 h. Triethylammonium acetate buffer (0.1 M, pH 7.0) was added, and MeOH was removed in vacuo. The crude oligonucleotides in the solution were purified using Sep-Pak^®^ Plus C18 cartridges (Waters), followed by reversed-phase HPLC (Waters XBridge^TM^ Prep Shield RP18, 5 μm, 10 × 50 mm). The compositions of the oligonucleotides, **ON1** and **ON2,** were confirmed by ESI-TOF-MS analysis. The deconvoluted ESI-TOF-MS data [M] for **ON1** and **ON2** are as follows: **ON1**, found 3905.10 (calcd. 3904.51); **ON2**, found 4430.50 (calcd. 4429.91).

Typical procedure for post-synthetic modification of **ON1** and **ON2**: A solution of **ON1** (final concentration—20 μM) and MeNH_2_ (final concentration—0.5 M) in H_2_O (100 μL) was maintained at 30 °C for 4 h. After the addition of AcOH (20 μL), the mixture was diluted with 0.1 M triethylammonium acetate buffer (pH 7.0) and purified by reversed-phase HPLC (Waters XBridge^TM^ Prep Shield RP18, 2.5 μm, 4.6 × 50 mm) to give **ON3b** in 86% yield. The isolated yield was calculated using the absorbance at 260 nm, which was measured on a NanoDrop 2000 spectrophotometer. The compositions of the oligonucleotides, **ON3a**–**i** and **ON4a**–**i,** were confirmed by ESI-TOF-MS analysis. The deconvoluted ESI-TOF-MS data [M] for **ON3a**–**i** and **ON4a**–**i**: **ON3a**, found 3737.90 (calcd. 3737.47); **ON3b**, found 3751.80 (calcd. 3751.50); **ON3c**, found 3766.00 (calcd. 3765.53); **ON3d**, found 3791.80 (calcd. 3791.57); **ON3e**, found 3807.00 (calcd. 3806.58); **ON3f**, found 3781.00 (calcd. 3780.54); **ON3g**, found 3795.00 (calcd. 3794.56); **ON3h**, found 3866.10 (calcd. 3865.69); **ON3i**, found 3909.20 (calcd. 3908.76); **ON4a**, found 4263.30 (calcd. 4262.88); **ON4b**, found 4277.30 (calcd. 4276.91); **ON4c**, found 4291.20 (calcd. 4290.93); **ON4d**, found 4317.50 (calcd. 4316.97); **ON4e**, found 4332.60 (calcd. 4331.99); **ON4f**, found 4306.30 (calcd. 4305.95); **ON4g**, found 4320.50 (calcd. 4319.97); **ON4h**, found 4391.70 (calcd. 4391.10); and **ON4i**, found 4434.70 (calcd. 4434.17).

### 3.3. UV-Melting Experiment

In the duplex-forming experiment, the synthesized oligonucleotides and ssRNA or ssDNA were dissolved in a 10 mM sodium phosphate buffer (pH 7.0) containing 200 mM NaCl to give a final concentration of 2.5 μM. In the triplex-forming experiment, the synthesized oligonucleotides and hairpin dsDNA were dissolved in a 10 mM sodium phosphate buffer (pH 7.0) containing 200 mM KCl and 5 mM MgCl_2_ to give a final concentration of 1.5 μM. The samples were annealed in boiling water followed by slow cooling to 5 °C. The melting profiles were recorded at 260 nm from 20 °C to 80 °C for ssRNA and ssDNA, and from 15 °C to 90 °C for dsDNA at a scan rate of 0.5 °C/min. The two-point average method was employed to obtain the *T*_m_ values, and the final values were determined by averaging three independent measurements, which were accurate within a 1 °C range.

## 4. Conclusions

A thymidine phosphoramidite of 2′-*N*-pentafluorophenoxycarbonyl-2′-amino-LNA was successfully synthesized and introduced into oligonucleotides. Treatment of the oligonucleotides with various amines could efficiently produce modified oligonucleotides containing the corresponding 2′-urea-LNA derivatives. This method could also be applied to the modification of resin-attached oligonucleotides. Moreover, the UV-melting experiments of the modified oligonucleotides suggested that 2′-urea-LNA—analogous to 2′-amino-LNA and its *N*-alkyl, *N*-acyl, and *N*-alkoxycarbonyl derivatives—could be promising as a chemical modification moiety of an oligonucleotide. Therefore, the post-synthetic modification using 2′-*N*-pentafluorophenoxycarbonyl-2′-amino-LNA allows for the exploration of high-performance oligonucleotides containing 2′-urea-LNA derivatives. Moreover, if the developed *O*-pentafluorophenyl carbamate unit was inserted into other sites of oligonucleotides, like the 5′-terminus or the nucleobase, the oligonucleotide could be post-synthetically linked to a functional molecule via a urea linkage.

## Figures and Tables

**Table 1:** Isolated yields of oligonucleotides containing 2′-urea-LNA derivatives.

Substrates	Products	Isolated Yield	Substrates	Products	Isolated Yield
**ON1** ^1^	**ON3a**	76%	**ON2** ^1^	**ON4a**	67%
**ON1** ^2^	**ON3b**	86%	**ON2** ^2^	**ON4b**	69%
**ON1** ^2^	**ON3c**	68%	**ON2** ^2^	**ON4c**	46%
**ON1** ^3^	**ON3d**	84%	**ON2** ^3^	**ON4d**	82%
**ON1** ^3^	**ON3e**	80%	**ON2** ^3^	**ON4e**	79%
**ON1** ^3^	**ON3f**	95%	**ON2** ^3^	**ON4f**	70%
**ON1** ^3^	**ON3g**	94%	**ON2** ^3^	**ON4g**	77%
**ON1** ^3^	**ON3h**	94%	**ON2** ^3^	**ON4h**	76%
**ON1** ^3^	**ON3i**	83%	**ON2** ^3^	**ON4i**	73%

^1^ Conditions: 10 M NH_3_ aq., 30 °C, 24 h. ^2^ Conditions: 0.5 M amine aq., 30 °C, 4 h. ^3^ Conditions: 0.5 M amine aq., 30 °C, 24 h.

**Table 2:** *T*_m_ values of duplexes containing modified oligonucleotides ^1^.

Oligonucleotides ^2^		*T*_m_ (Δ*T*_m_^3^) with ssDNA	*T*_m_ (Δ*T*_m_^3^) with ssRNA
**ON3a**	X = NH_2_	54 °C (+3 °C)	58 °C (+6 °C)
**ON3b**	X = NHMe	53 °C (+2 °C)	58 °C (+6 °C)
**ON3c**	X = NMe_2_	52 °C (+1 °C)	57 °C (+5 °C)
**ON3d**	X = pyrrolidin-1-yl	52 °C (+1 °C)	57 °C (+5 °C)
**ON3e**	X = piperazin-1-yl	54 °C (+3 °C)	57 °C (+5 °C)
**ON3f**	X = NH(CH_2_)_2_NH_2_	55 °C (+4 °C)	57 °C (+5 °C)
**ON3g**	X = NH(CH_2_)_3_NH_2_	55 °C (+4 °C)	58 °C (+6 °C)
**ON3h**	X = NH(CH_2_)_3_NH(Me)(CH_2_)_3_NH_2_	54 °C (+3 °C)	57 °C (+5 °C)
**ON3i**	X = NH(CH_2_)_3_N[(CH_2_)_3_NH_2_]_2_	55 °C (+4 °C)	55 °C (+3 °C)
**ON5**	X = OMe	52 °C (+1 °C)	58 °C (+6 °C)
**ON6**	2′-amino-LNA	52 °C (+1 °C)	58 °C (+6 °C)
**ON7**	natural	51 °C	52 °C

^1^ Conditions for duplexes: 10 mM sodium phosphate buffer (pH 7.0), 200 mM NaCl, and 2.5 µM of each oligonucleotide. The sequences of ssDNA and ssRNA are 5′-d(ACGAGAACATCC)-3′ and 5′-r(ACGAGAACAUCC)-3′, respectively. ^2^ The sequences and structures of modified oligonucleotides used are shown in Scheme 2 and Figure 3. ^3^ The change in *T*_m_ values relative to natural **ON7**.

**Table 3:** *T*_m_ values of triplexes containing modified oligonucleotides ^1^.

Oligonucleotides ^2^		*T*_m_ (Δ*T*_m_^3^)
**ON4a**	X = NH_2_	38 °C (+7 °C)
**ON4b**	X = NHMe	36 °C (+5 °C)
**ON4c**	X = NMe_2_	38 °C (+7 °C)
**ON4d**	X = pyrrolidin-1-yl	31 °C (0 °C)
**ON4e**	X = piperazin-1-yl	34 °C (+3 °C)
**ON4f**	X = NH(CH_2_)_2_NH_2_	35 °C (+4 °C)
**ON4g**	X = NH(CH_2_)_3_NH_2_	35 °C (+4 °C)
**ON4h**	X = NH(CH_2_)_3_NH(Me)(CH_2_)_3_NH_2_	35 °C (+4 °C)
**ON4i**	X = NH(CH_2_)_3_N[(CH_2_)_3_NH_2_]_2_	35 °C (+4 °C)
**ON8**	X = OMe	36 °C (+5 °C)
**ON9**	2′-amino-LNA	37 °C (+6 °C)
**ON10**	natural	31 °C

^1^ Conditions for triplexes: 10 mM sodium phosphate buffer (pH 7.0), 200 mM KCl, 5 mM MgCl_2_, and 1.5 µM of each oligonucleotide. The sequence of dsDNA is 5′-d(GGCAGAAGAAAAAGAGACGC)-spacer18-d(GCGTCTCTTTTTCTTCTGCC)-3′ (spacer18 = hexaethylene glycol linker). ^2^ The sequences and structures of modified oligonucleotides used are shown in Scheme 2 and Figure 3. ^3^ The change in *T*_m_ values relative to natural **ON10**.

## Supplementary Materials

The following are available online, Figure S1: An example of post-synthetic modification of **ON2**, Figure S2: ^1^H-NMR and ^13^C-NMR spectra of compound **2**, Figure S3: ^1^H-NMR and ^31^P-NMR spectra of compound **3**.
