# Supplementary material for: Synthesis of Oligonucleotides Containing 2′-N-alkylaminocarbonyl-2′-amino-LNA (2′-urea-LNA) Moieties Using Post-Synthetic Modification Strategy
Source: Molecules. 2020 Jan 15;25(2):346. doi: 10.3390/molecules25020346 (PMC7024358; doi:10.3390/molecules25020346)
Supplement: Supplementary file 1 [file molecules-25-00346-s001.pdf]

Supplementary Materials

Synthesis of oligonucleotides containing 2′-*N*-alkylaminocarbonyl-2′-amino-LNA (2′-urea-LNA) moieties using post-synthetic modification strategy

Shoko Yamashita [1,†], Kodai Nishida [1,†], Takashi Osawa [1,2], Ayumi Nakanishi [1], Yuta Ito [1] and Yoshiyuki Hari [1,*]

[1] Faculty of Pharmaceutical Sciences, Tokushima Bunri University, Nishihama, Yamashiro-cho, Tokushima 770-8514, Japan

[2] Graduate School of Pharmaceutical Sciences, Osaka University, 1-6 Yamadaoka, Suita 565-0871, Japan

***** Correspondence: hari@ph.bunri-u.ac.jp

[†] These authors contributed equally to the work.

Table of contents

1. An example of post-synthetic modification of **ON2** (Figure S1) ……………………….……………S2

2. ^{1}H and ^{13}C NMR spectra of compound **2** (Figure S2)…………………….…………………. S3

3. ^{1}H and ^{31}P NMR spectra of compound **3** (Figure S3)…………………….…………………. S4

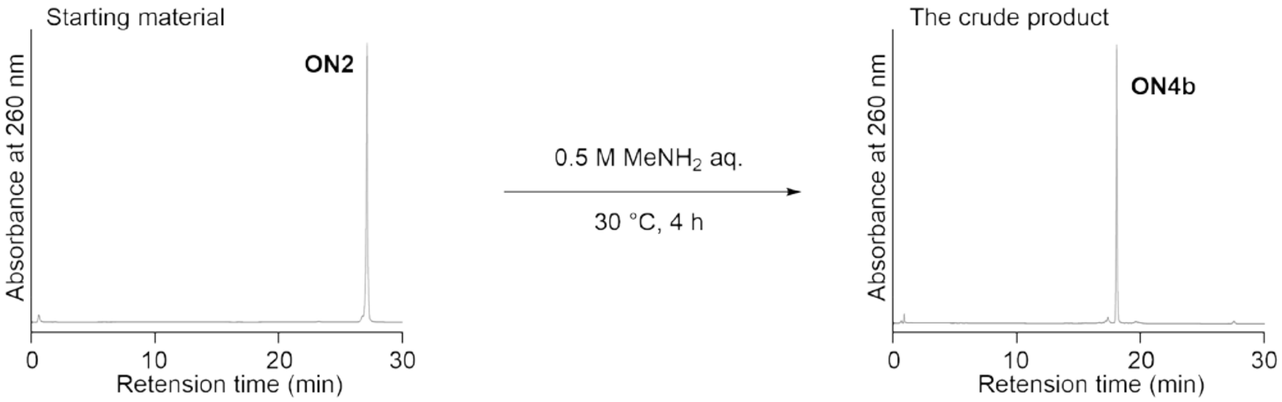

Figure S1. An example of post-synthetic modification of **ON2**.

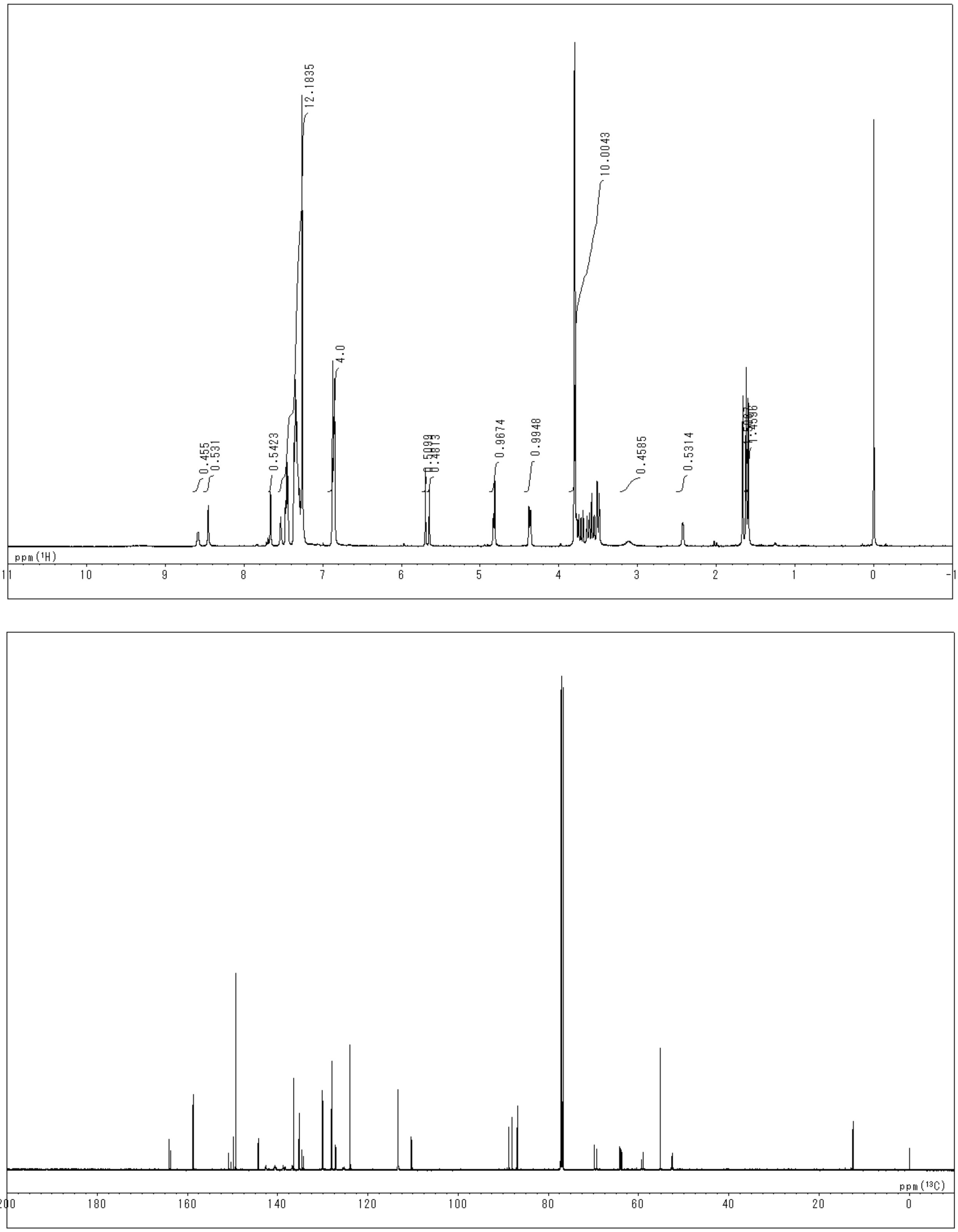

Figure S2. ^{1}H and ^{13}C NMR spectra of compound **2**

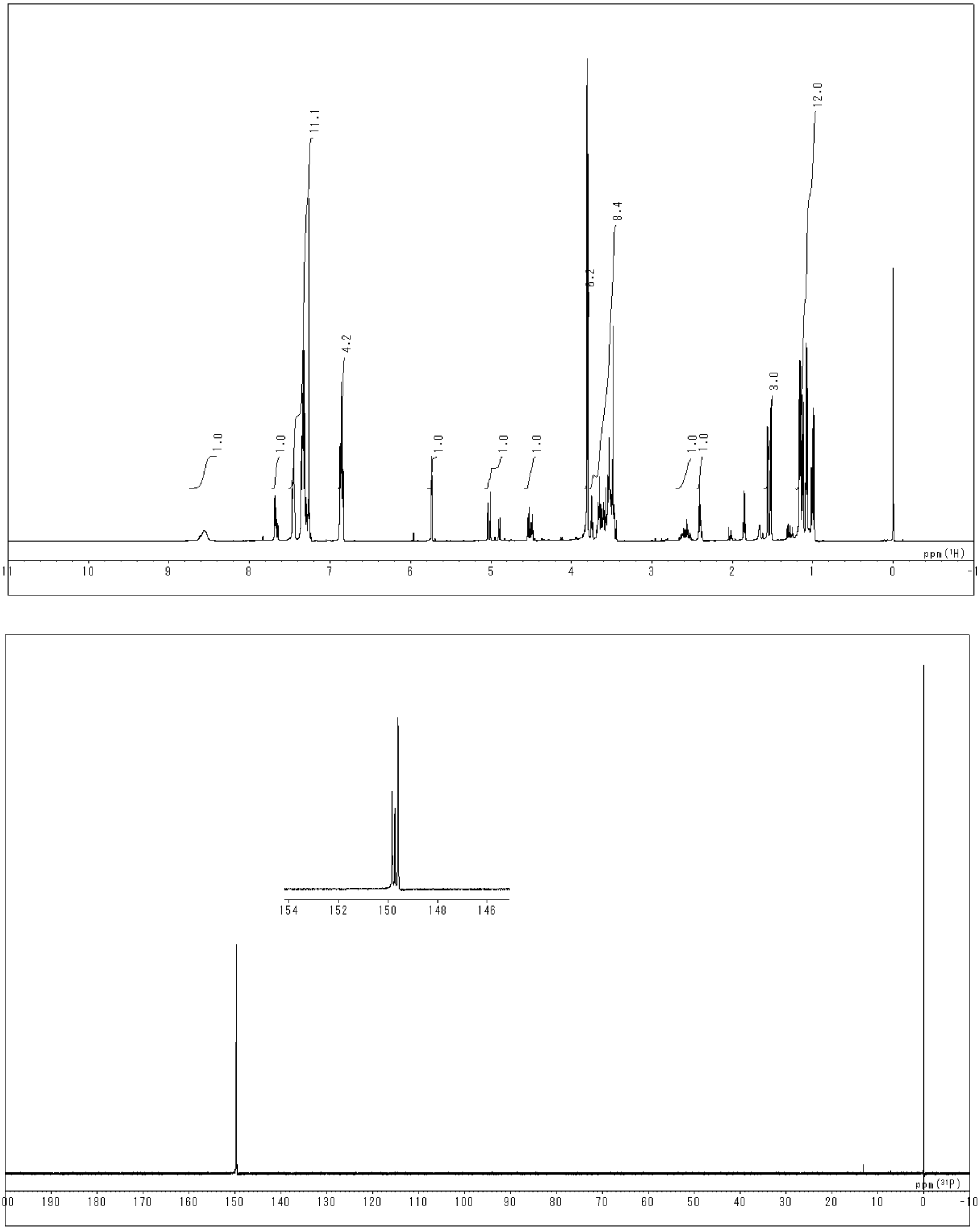

Figure S3. ^{1}H and ^{31}P NMR spectra of compound **3**
